# The GNAQ T96S Mutation Affects Cell Signaling and Enhances the Oncogenic Properties of Hepatocellular Carcinoma

**DOI:** 10.3390/ijms22063284

**Published:** 2021-03-23

**Authors:** Eugene Choi, Sung Jean Park, Gunhee Lee, Seung Kew Yoon, Minho Lee, Suk Kyeong Lee

**Affiliations:** 1 Department of Medical Life Sciences, Department of Biomedicine & Health Sciences, College of Medicine, The Catholic University of Korea, Seoul 06591, Korea; lightnsalt@catholic.ac.kr; 2 College of Pharmacy and Gachon Institute of Pharmaceutical Sciences, Gachon University, Incheon 21936, Korea; psjnmr@gachon.ac.kr; 3 Precision Medicine Research Center, Integrated Research Center for Genome Polymorphism, Department of Microbiology, College of Medicine, The Catholic University of Korea, Seoul 06591, Korea; gunheelee@catholic.ac.kr; 4 Department of Internal Medicine, Seoul St. Mary’s Hospital, College of Medicine, The Catholic University of Korea, Seoul 06591, Korea; yoonsk@catholic.ac.kr; 5 Department of Life Science, Dongguk University-Seoul, Ilsandong-gu, Goyang-si 10326, Gyeonggi-do, Korea; MinhoLee@dgu.edu

**Keywords:** hepatocellular carcinoma, guanine nucleotide-binding protein G(q) subunit alpha, helical domain, somatic mutation, mitogen-activated protein kinase (MAPK) signaling

## Abstract

Hepatocellular carcinoma (HCC), the most common malignant tumor in the liver, grows and metastasizes rapidly. Despite advances in treatment modalities, the five-year survival rate of HCC remains less than 30%. We sought genetic mutations that may affect the oncogenic properties of HCC, using The Cancer Genome Atlas (TCGA) data analysis. We found that the GNAQ T96S mutation (threonine 96 to serine alteration of the Gαq protein) was present in 12 out of 373 HCC patients (3.2%). To examine the effect of the GNAQ T96S mutation on HCC, we transfected the SK-Hep-1 cell line with the wild-type or the mutant GNAQ T96S expression vector. Transfection with the wild-type GNAQ expression vector enhanced anchorage-independent growth, migration, and the MAPK pathways in the SK-Hep-1 cells compared to control vector transfection. Moreover, cell proliferation, anchorage-independent growth, migration, and the MAPK pathways were further enhanced in the SK-Hep-1 cells transfected with the GNAQ T96S expression vector compared to the wild-type GNAQ-transfected cells. In silico structural analysis shows that the substitution of the GNAQ amino acid threonine 96 with a serine may destabilize the interaction between the regulator of G protein signaling (RGS) protein and GNAQ. This may reduce the inhibitory effect of RGS on GNAQ signaling, enhancing the GNAQ signaling pathway. Single nucleotide polymorphism (SNP) genotyping analysis for Korean HCC patients shows that the GNAQ T96S mutation was found in only one of the 456 patients (0.22%). Our data suggest that the GNAQ T96S hotspot mutation may play an oncogenic role in HCC by potentiating the GNAQ signal transduction pathway.

## 1. Introduction

Liver cancer is globally the sixth-most-common type of cancer and the fourth-most-common cause of cancer mortality [1]. Single nucleotide polymorphisms (SNPs) in liver cancer have been studied as a cancer risk factor contributing to poor prognosis in patients [2,3,4]. The somatic mutation S1113R of the phosphatidylinositol-3,4,5-trisphosphate dependent Rac exchange factor 2 (*PREX2*) gene has been shown to promote migration and proliferation and activate the AKT pathway in hepatocellular carcinoma (HCC) [5]. Meanwhile, the polymorphisms of UDP-glucuronosyltransferase 1A enzymes contribute to HCC development in vivo [6], suggesting the potential of single amino acid substitutions as a biomarker for liver cancer.

The G protein plays an essential role in cellular signal transduction. The somatic mutation R201H in *gnas* (encoding Gαs) promotes pancreatic tumorigenesis in murine models that recapitulates human intraductal papillary mucinous neoplasm [7]. Moreover, the Arg-200 mutation in *GNA13* (encoding Gα13) has been proposed as a putative driver of bladder cancer [8]. Guanine nucleotide-binding protein G(q) subunit alpha (*GNAQ*, encoding Gαq), *GNAS*, and *GNA14* (encoding Gα14) mutations were also found in hepatic small vessel neoplasm and intrahepatic cholangiocarcinoma in the liver, although their roles have not been elucidated [9,10]. Thus, functional studies on the effects of G protein mutations in liver cancer are needed. 

Gαq is a component of a heterotrimeric protein that binds with guanosine triphosphate (GTP) and cycles between active and inactive states by hydrolyzing GTP to guanosine diphosphate (GDP). The Gαq protein consists of 359 amino acids and contains two primary domains: the GTPase domain and the helical domain. Within the GTPase domain are three switch regions [11], which are the primary contact sites for the Gβγ subunit (Switch II), the regulator of G protein signaling (RGS), and the effector. The helical domain consists entirely of α helices and is characterized by a unique sequence for each Gα protein [12]. Gαq is one of the most critical modulators and transducers of various transmembrane signaling systems. It mediates many signaling pathways, including the mitogen-activated protein kinase (MAPK) signaling pathway [13,14,15]. 

When the whole genome sequence data for 11,119 cancer tissues of 41 cancer types were analyzed, common recurrent hotspot mutations of several genes were identified in various cancers [16]. Among the recurrent mutations in *GNAQ*, new hotspot mutations were identified at codons 96 and 48, in addition to the already-known mutations at codons 183 and 209. The codon 96 mutation is expressed in the helical domain of Gαq and is a missense mutation in which nucleotide 286 adenine is changed to thymine, resulting in amino acid 96 changing from threonine to serine (hereafter referred to as “T96S”) [16].

The GNAQ T96S mutation has been reported in various diseases. In gastric cancer (GC), the GNAQ T96S mutation was predicted to bind with the highly frequent HLA allele as a tumor-specific neoantigen, and suggested as a potential target for T cell immunotherapy [17]. GNAQ T96S was observed as a rare and frequent mutation in prostate cancer and non-small-cell lung cancer (NSCLC), respectively [13,18]. The GNAQ T96S mutation was also detected in a three-year-old boy with diffuse bone and soft tissue angiomatosis [19]. Besides, In natural killer/T cell lymphoma (NKTCL), the GNAQ T96S mutation shows strong dominant-negative effects in both in vitro and in vivo experiments [14]. However, few studies report the role of *GNAQ* T96S in HCC.

We investigated the role of the *GNAQ* T96S mutation in HCC by analyzing the effect of *GNAQ* T96S overexpression on various phenotypes and intracellular signaling pathways in HCC cells.

## 2. Results

### 2.1. The Cancer Genome Atlas (TCGA) Genotype Data in Liver Cancer Patients with HCC

We investigated the liver hepatocellular carcinoma (LIHC) category in the Broad Institute’s Genome Data Analysis Center (GDAC; https://gdac.broadinstitute.org, accessed date: 4 September 2018). We found the GNAQ T96S somatic mutation in samples of 12 patients out of 373 HCC patients (3.2%). All patients with the mutation were male and had a heterozygous genotype on the locus. Four of the 12 patients were White; eight were Asian. The age range was quite wide (18–80, average 54.83, Supplementary Data S1).

We also investigated whether GNAQ T96S was associated with other liver cancer risk factors, including sex, alcohol consumption, alpha-1 antitrypsin deficiency, hemochromatosis, hepatitis B, hepatitis C, and nonalcoholic fatty liver disease. We found no association with other liver cancer risk factors except sex (Table 1, Supplementary data S2). 

### 2.2. Sequencing and Overexpression of the GNAQ T96S Hotspot Mutation in SK-Hep-1 Cells

**Figure 1:**
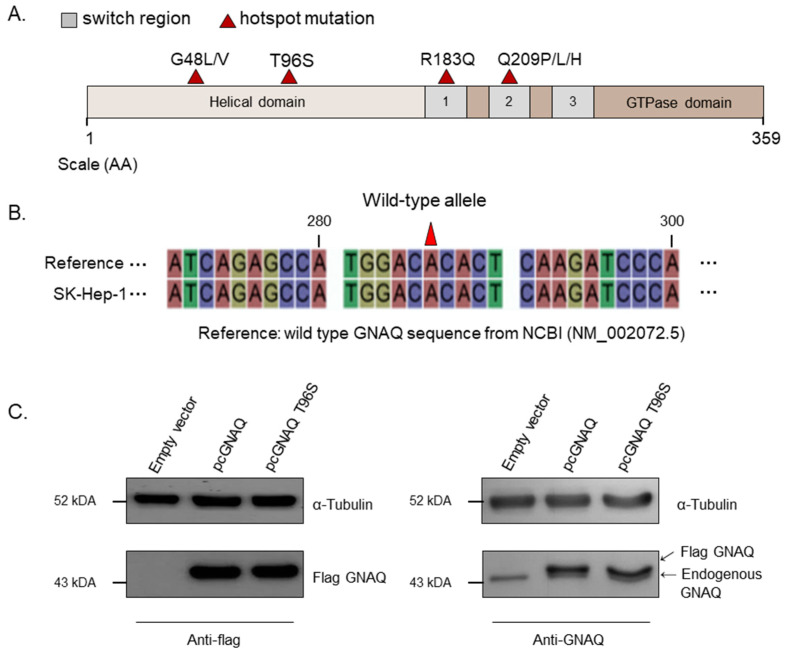
*GNAQ* T96S mutation. (**A**) Four hotspot mutations mapped in a schematic diagram of the GNAQ protein sequence (amino acids 1 to 359). Helical domain (beige), GTPase domain (brown), switch regions 1–3 (gray), and hotspot mutations (red triangles) are indicated. (**B**) The GNAQ sequence in the SK-Hep-1 cell line. The GNAQ sequence of SK-Hep-1 was analyzed and compared to the wild-type GNAQ reference sequence from the National Center for Biotechnology Information (NCBI; NM_002072.5). (**C**) Overexpression of the wild-type or the T96S mutant GNAQ. The control vector, pcGNAQ, or pcGNAQ T96S was transfected into SK-Hep-1 cells. After 24 h, a Western blot was performed to analyze the level of GNAQ protein using anti-Flag (left) or anti-GNAQ (right) antibodies in SK-Hep-1. Anti-α-tubulin antibody was used for normalization.

Hotspot mutations located in GNAQ are marked in a schematic diagram of GNAQ (Figure 1A). GNAQ T96S is located in the helical domain of GNAQ, unlike R183Q or Q209P/L/H, which are located in the switch regions. We performed GNAQ sequencing to test whether the SK-Hep-1, HepG2, Hep3B, and SNU-387 cell lines—which are normally used in HCC cell experiments—have the wild-type GNAQ. The GNAQ sequence of every cell line was identical (data not shown), with the NM_002072.5 GNAQ wild-type reference sequence of the National Center for Biotechnology Information (NCBI); we selected the SK-Hep-1 cell line to use for later experiments (Figure 1B). Western blot analysis for GNAQ was carried out following transfection with the vectors for control, pcGNAQ, or pcGNAQ T96S to SK-Hep-1. As expected, flag-tagged GNAQ was not detected in the control vector-transfected cells using the anti-FLAG antibody. In contrast, FLAG GNAQ and FLAG GNAQ T96S were detected at high levels in cells transfected with pcGNAQ and pcGNAQ T96S, respectively. The expression levels of FLAG GNAQ and FLAG GNAQ T96S were comparable.

When GNAQ expression was detected using anti-GNAQ antibody, endogenously expressed GNAQ was detected in the cells transfected with the control vector, pcGNAQ, and pcGNAQ T96S vectors. In addition, overexpressed FLAG GNAQ and FLAG GNAQ T96S were also detected using anti-GNAQ antibodies in cells transfected with pcGNAQ and pcGNAQ T96S, respectively. The expression level of FLAG GNAQ was approximately five times higher than the endogenously expressed GNAQ level; the size of FLAG GNAQ was slightly bigger than the endogenous GNAQ, due to FLAG tagging (Figure 1C). 

### 2.3. Effect of GNAQ and GNAQ T96S on Cell Proliferation, Anchorage-Independent Growth, and Migration in SK-Hep-1 Cells

**Figure 2:**
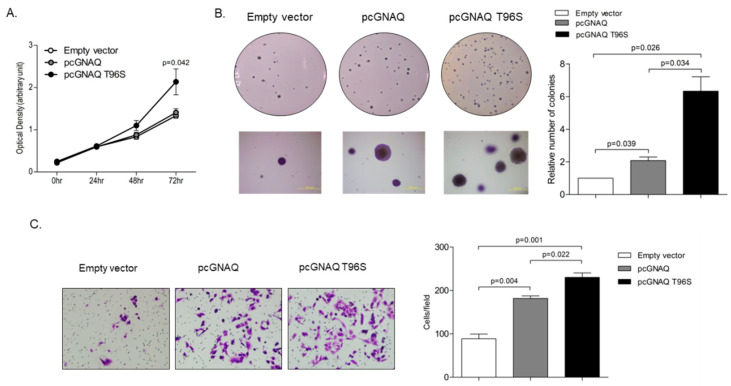
Effect of the GNAQ T96S mutation on cell proliferation, anchorage-independent growth, and migration. SK-Hep-1 cells were transfected with the control vector, pcGNAQ, or pcGNAQ T96S. (**A**) Effect of the GNAQ T96S mutation on cell growth. To measure SK-Hep-1 cell proliferation, 20 µL of 3-(4,5-dimethylthiazol-2-yl)-2,5-diphenyltetrazolium bromide (MTT) solution was added to each well immediately after transfection and every 24 h after transfection for three days. Absorbance at 540 nm was analyzed via SoftMax apparatus. Error bars indicate SD (*n* = 3). (**B**) Effect of the GNAQ T96S mutation on anchorage-independent cell growth. The cells were harvested 24 h after transfection and seeded in agar. Cells were cultured for three weeks in a 37 °C CO_2_ incubator and then observed. Upper panels show pictures taken with a camera. Lower panels show images observed with microscope IX70 through a ×40 objective (left). Similar experiments were carried out three times independently. Each value represents the mean SD of all three experiments (right). (**C**) Transwell migration assay. The cells were harvested 24 h after transfection and seeded in the upper chamber. After 48 h, the migrated cells were stained and observed with a microscope IX70 through a ×200 objective (left). Similar experiments were carried out three times independently. Each value represents the mean SD of all three experiments (right).

To investigate the effect of GNAQ and GNAQ T96S overexpression on cell proliferation and anchorage-independent growth, we performed a 3-(4,5-dimethylthiazol-2-yl)- 2,5-diphenyltetrazolium bromide (MTT) assay and a soft agar colony formation assay. The MTT assay revealed that SK-Hep-1 cell proliferation was not affected when the cells were transfected with the GNAQ wild-type overexpression vector, compared to transfection with the empty control vector. In contrast, proliferation was 1.5-times higher with GNAQ T96S transfection than with control vector transfection, when analyzed 72 h after transfection (Figure 2A). A soft agar colony formation assay showed that anchorage-independent growth of SK-Hep-1 cells was two times higher in cells transfected with pcGNAQ compared to control cells. Remarkably, pcGNAQ T96S transfection increased anchorage-independent growth six times higher, compared to control vector transfection (Figure 2B).

For HCC, metastasis is the most important biological characteristic and a major cause of treatment failure [20]. Therefore, we next investigated whether the GNAQ and GNAQ T96S overexpression affect HCC cell migration, which is related to cancer metastasis. Based on results from a transwell migration assay, cell migration seems to be increased 200% in cells transfected with pcGNAQ, compared to the control vector-transfected cells. Moreover, pcGNAQ T96S transfection caused enhanced cell migration up to 260% higher in a transwell migration assay relative to the control vector transfection (Figure 2C).

### 2.4. GNAQ and GNAQ T96S Enhance ERK Signaling in SK-Hep-1 Cells 

**Figure 3:**
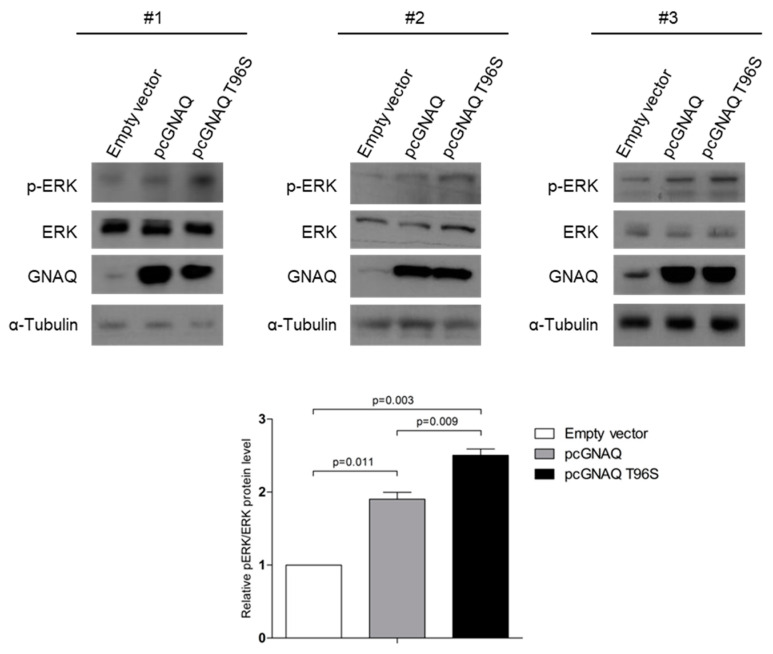
Increased ERK signaling due to GNAQ T96S mutation. SK-Hep-1 cells were transfected with the control vector, pcGNAQ, or pcGNAQ T96S. Western blot was carried out using three independently transfected cell sets to detect total and phosphorylated forms of ERK. Anti-ERK (1:500), anti-pERK (1:500), and anti-GNAQ (1:500) antibodies were used. Comparable loading amounts were confirmed by detection with anti-α-tubulin (1:2000) antibody. Phosphorylated ERK levels were normalized by the total ERK levels in the plot shown at the lower panel.

As elevated MAPK signals have been reported in human NKTCL samples containing the mutant GNAQ T96S, compared to those having the wild-type GNAQ [14], we performed a Western blot to analyze the changes in levels of pERK following GNAQ T96S transfection in HCC. The pERK levels were increased, while the total ERK levels were not affected in the cells transfected with pcGNAQ compared to those transfected with the control vector. In addition, pERK levels were even higher in pcGNAQ T96S-transfected cells compared to the pcGNAQ and control vector-transfected cells (Figure 3). 

### 2.5. Screening for the GNAQ T96S (rs753716491) Mutation in the Korean HCC Population

**Figure 4:**
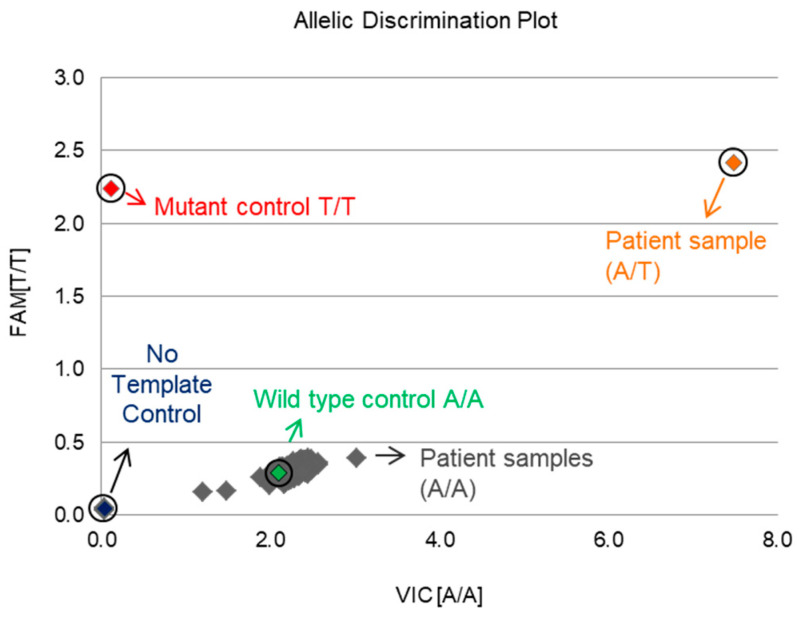
Screening for the GNAQ T96S (rs753716491) mutation in 456 Korean patients. A single nucleotide polymorphism (SNP) assay was performed to determine the frequency of the GNAQ T96S mutation in Korean liver cancer patients. The cancer tissues of 456 Korean liver cancer patients were collected, and DNA was extracted. DNA samples were genotyped with an rs753716491 probe in the Taqman real-time polymerase chain reaction (PCR) system.

To investigate the frequency of the T96S mutation in Korean patients, we performed an SNP assay with DNA samples extracted from 316 male and 140 female Korean HCC tissues. We used pcGNAQ and pcGNAQ T96S as the controls for the wild-type allele (A/A) and mutant allele (T/T), respectively. An NTC (no template control) sample containing no DNA was used as a negative control. Out of 456 samples, only 1 case had a heterozygous mutant allele (A/T); the remaining 455 cases had the wild-type allele (A/A). The frequency of the T96S mutation was relatively low (about 0.22%) in Korean liver cancer patients, compared to 3.2% in TCGA liver cancer patients. In addition, all TCGA liver cancer patients showing the T96S mutation were male, although the only Korean patient with the T96S mutation was female (Figure 4).

### 2.6. In Silico Structural Analysis of the GNAQ T96 and RGS2 Protein Complex

**Figure 5:**
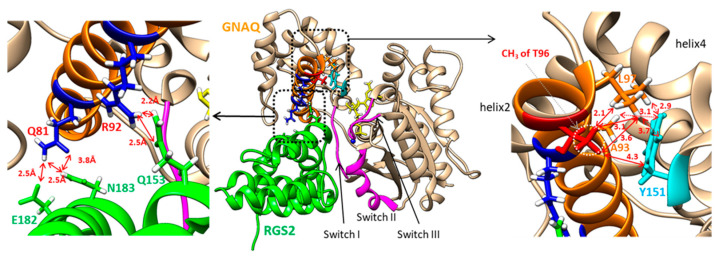
The complex structure (PDB ID: 4EKD) of GNAQ (tan) and RGS2 (green). The regions of Switches I–III are depicted in pink. Helix 2 of GNAQ is colored in orange. The dotted rectangles in the center are enlarged in the left or right panels. The left panel shows the hydrogen bonding network between the helical domain of GNAQ and RGS2. The identification of hydrogen bonding was calculated using the software University of California, San Francisco (UCSF) Chimera. The right panel shows the hydrophobic network between helix 2 and helix 4 of GNAQ. The side chain of T96 is depicted in red. The side-chain atoms of Tyr151 are shown in cyan. The solid-line arrows represent the distances between the methyl protons of T96 and the other atoms. The dotted line arrows show the distance between the side chain of Tyr151 and the side chain of Ala93 and Leu97. The distance unit, Å, is removed for clear presentation.

Based on the complex structure of GNAQ and RGS2, it is clear that helix 2—including T96 in the helical domain of GNAQ—closely contacts helix 7 of RGS2, as shown in Figure 5. In the complex of RGS2 and GNAQ, helix 4 of RGS2 binds to Switch I, while helix 6 of RGS2 contacts Switches II and III [21]. Figure 5 shows that the oxygens of the side-chain carboxyl group of Glu182 in RGS2 form the hydrogen-bonding network with the hydrogen of the ε-ammonium group of Lys77 and with the hydrogen of the side-chain amide of Gln81 in helix 2 of GNAQ. In addition, the side-chain amide proton of Asn183 closely locates toward the side-chain carbonyl oxygen of Gln81 in GNAQ. Interestingly, T96 at the end of helix 2 in GNAQ adopts a hydrophobic core with Ala93, Leu97, and Tyr151. The side-chain methyl protons of T96 and the side-chain methyl protons of Leu97 form close contact within about 3 Å, and the methyl protons of T96 also locate near the side-chain protons of Tyr151 in helix 4 of GNAQ. The methyl protons of Ala93 contribute the hydrophobic contact with T96 and Tyr151. The atomic distances of all these hydrophobic contacts range from 2 Å to 5 Å (Figure 5). 

## 3. Discussion

We found the GNAQ T96S mutation in liver cancer through TCGA data analysis. Transfection of the GNAQ wild-type expression vector increased anchorage-independent growth, migration, and phosphorylated forms of ERK in HCC cells compared to the transfection of the control vector. In addition, the GNAQ T96S overexpression increased cell proliferation as well as enhanced oncogenic phenotypes and ERK phosphorylation compared to the wild-type GNAQ overexpression. 

The GNAQ T96S mutation in NKTCL promotes tumorigenesis through a dominant-negative function that suppresses wild-type GNAQ, which inhibits MAPK signaling [14]. Our results also indicate that the GNAQ T96S mutation promotes tumorigenesis and activation of the ERK pathways in liver cancer cells. The role of the wild-type GNAQ differs depending on the carcinomas. In GC, GNAQ knockdown inhibits cell growth and the MAPK pathway, suggesting that GNAQ functions as an oncogene [22]. In contrast, GNAQ shows a tumor-suppressive effect in both NK and NKTCL cells [14], as well as in NSCLC cells [13], through regulating the AKT and ERK pathways. In melanoma, no effects have been observed in vitro or in vivo [23,24]. The role of wild-type GNAQ and GNAQ T96S in liver cancer has not yet been clearly understood. In this study, the overexpression of wild-type GNAQ in SK-Hep-1 cells increased anchorage-independent growth, migration, and onco-signaling pathways compared to the control vector-transfected cells. In contrast, proliferation was not affected by wild-type GNAQ. Since carcinogenic phenotypes such as growth and migration are not necessarily positively correlated [25], the role of wild-type GNAQ in liver cancer warrants further investigation using more cells and patients.

The sample of TCGA liver cancer patients that we analyzed includes 252 men and 151 women (total 373 patients, M:F = 1:0.6), while the Korean HCC patients analyzed in this study include 316 males and 140 females (total 456 patients, m:f = 1:0.44). The frequency of patients possessing the GNAQ T96S mutation was far less in Korean patients (0.22%) than in TCGA patients (3.2%). Different mutation frequencies for the same gene have been found depending on ethnicity in other studies, too. In TCGA HCC patients, the mutation frequencies of TP53 and RB1 were 43% and 19% in Asian Americans and 21% and 2% for those with White Americans, respectively [26,27]. Because GNAQ T96S was found only in male liver cancer patients in the TCGA study, we initially theorized that the GNAQ T96S mutation might be a male-specific biomarker for liver cancer. However, when we tested the samples from Korean liver cancer patients, the only patient who showed the GNAQ T96S mutation was female. Therefore, it is unlikely that GNAQ T96S is a sex-specific biomarker for liver cancer patients. Notably, the mutation found in TCGA was a somatic mutation, indicating that the mutation was not detected in matched normal cases. However, we genotyped only tumor samples for the Korean HCC patients since we could not collect the matched normal tissue samples. Therefore, our finding that one tissue sample of a female HCC patient had the mutation should be interpreted carefully. Further clarification of the effects of the GNAQ T96S mutation in a female HCC cell line such as SNU-387 may provide additional ideas for the sex-dimorphism of the GNAQ T96S mutation. 

As shown in supplementary data 1, one of the patients is 18-years-old while the others are over 40. This patient is an extreme outlier and further study is warranted to clarify whether GNAQ T96S has affected this patient in a mechanistically similar way compared to other patients.

The GNAQ T96S mutation is located in the helical domain of the protein. The helical domain of the Gα subunit has not received significant attention, although recent studies have begun to elucidate its function. A unique sequence of the helical domain in each Gα subunit may confer binding specificity for various proteins [12]. Some mutations in the Gαq helical domain decrease the binding affinity of Gα for interacting proteins [28], suggesting that amino acid substitution in the Gαq helical domain may affect protein-protein interactions. Essentially, the interaction of GNAQ with other signal proteins occurs through the contact sites named Switches I–III, which are located between the helical domain and the GTPase domain of GNAQ [11,12]. The loop regions of PLCβ3, as well as the C-terminal helix, form the major binding interface with Switches I–III of GNAQ [29]. Helix 4 of G protein-coupled receptor (GPCR) kinase 2 contacts Switch II of GNAQ [30]. Switches II and III also play important roles in the complexation of guanine nucleotide exchange factor 25 [31]. However, none of these complexes of GNAQ (except the RGS2 complex) use the helical domain of GNAQ for their binding. The RGS2 protein has been shown to inhibit the ERK signaling pathways by binding to Gαq [32]. Thus, it is possible that the increased activation of ERK by GNAQ T96S may result from reduced binding affinity between GNAQ T96S and RGS2, due to the helical domain mutation. Indeed, our in silico analysis results indicate that the hydrophobic contact of methyl protons of T96 may contribute to stabilizing the conformation and the orientation of helix 2 in the complex. The replacement of T96 with S96 may contribute to the formation of a less-stable helical network due to the absence of methyl protons. Thus, we suggest that increased activation of ERK by GNAQ T96S transfection may have occurred via a destabilized GNAQ-RGS2 complex.

In addition to HCC, the GNAQ T96S mutation was found in various cancers through the cBioPortal; these include pediatric pan-cancer [33], kidney cancer [34], lung cancer [35,36], myelodysplastic syndrome, gallbladder cancer [37], lymphoma-like diffuse giant B-cell lymphoma, pancreatic cancer [38], prostate adenocarcinoma [39], and cutaneous melanoma. The GNAQ codon 183 mutation was found in 80% (12 of 15) of patients with Sturge-Weber syndrome, a type of neurocutaneous disorder [40], while a mutation in GNAQ/11 codon 209 has been detected in more than 80% of uveal melanoma (UM) patients [24,41]. However, these GNAQ mutations were not observed among the HCC patients of the TCGA study. Thus, mutations at different sites in *GNAQ* seem to be associated with different tumors.

## 4. Materials and Methods

### 4.1. TCGA Analysis

To calculate the mutation allele frequency of GNAQ T96S, we selected a category (LIHC) in the Broad Institute’s Genome Data Analysis Center (GDAC). Next, we downloaded raw Mutation Annotation Files (MAFs) (Level 3). The downloaded data contained the mutation call files of 373 HCC patients. We calculated the occurrence of the mutation by investigating each MAF file and determined the mutation allele frequency. 

### 4.2. Cell Lines and Culture Conditions

SK-Hep-1 human HCC cell line was provided by Professor Sang Geon Kim (Dongguk University). HepG2, Hep3B, and SNU-387 cell lines were purchased from Korean Cell Line Bank. SK-HEP-1 and HepG2 were derived from White males aged 53 and 15-years-old, respectively. Hep3B was derived from an 8-year-old Black male, and SNU-387 was derived from a 41-year-old Asian female. SK-Hep-1, HepG2, and Hep3B were grown in Dulbecco’s modified Eagle’s medium (DMEM, Gibco, Carlsbad, CA, USA), while SNU-387 was grown in RPMI 1640 (Gibco, Grand Island, NY, USA). All media were supplemented with 10% fetal bovine serum (FBS), 1% penicillin-streptomycin (Gibco), and 0.1% Amphotericin B (Gibco). All cells were cultured at 37 °C and supplemented with 5% CO_2_. 

### 4.3. Clinical Samples

The HCC tumor tissues from 316 males and 140 females used for this study were provided by members of the Korea Biobank Network: Ajou University, Chungbuk National University, Jeju National University, Jeonbuk National University, and Pusan National University’s Hospital. This study was approved by the Songeui Medical Campus Institutional Review Board (IRB) of the Catholic University of Korea (IRB approval number: MC18SNSI0110). All biopsy samples were obtained after an additional IRB approval process at each institution. 

### 4.4. GNAQ Expression Vectors

The human GNAQ wild-type overexpression vector cloned in pcDNA3.1 was purchased from the cDNA Resource Center (Rolla, MO, USA). To introduce the T96S mutation into the wild-type GNAQ sequence, we used the EZchange site-directed mutagenesis kit (Enzynomics, Daejeon, South Korea). The following primers were used to produce the GNAQ T96S mutant vector: 5′-CAC TCA AGA TCC CAT ACA AGT ATG AGC-3′ (forward) and 5′-AGT CCA TGG CTC TGA TCA TGG C-3′ (Reverse). Polymerase chain reaction (PCR) conditions were 95 °C for 2 min, followed by 25 cycles at 95 °C for 30 s, 63 °C for 40 s, and 72 °C for 7 min. Flag tag was then introduced to the GNAQ wild-type overexpression vector to produce pcGNAQ, or added to the GNAQ T96S mutant vector to produce pcGNAQ T96S, using the EZchange site-directed mutagenesis kit. We used the following primers: 5′-GAT GAC GAC AAG ATG ACT CTG GAG TCC ATC ATG GCG TGC T-3′ (forward) and 5′-GTC TTT GTA GTC CAT GGT GGT ACC AAG CTT AAG TTT AAA CGC TAG CC-3′ (Reverse). The PCR conditions were 95 °C for 2 min, followed by 25 cycles at 95 °C for 30 s, 85 °C for 40 s, and 72 °C for 7 min. The sequences of pcGNAQ and pcGNAQ T96S were verified by DNA sequencing. 

### 4.5. Sequencing of GNAQ Exons in SK-Hep-1 Cells

The SK-Hep-1 cells were harvested and total RNA was extracted using the RNAisoplus (TaKaRa, Tokyo, Japan) reagent according to the manufacturer’s instruction. cDNA was synthesized using 1.5 µg total RNA, oligo(dT) primers (Macrogen, Seoul, Korea), and Moloney murine leukemia virus reverse transcriptase (Invitrogen, Carlsbad, CA, USA). The cDNA was amplified by PCR with the following primer set: 5′-GTG TGC GCG CTG TGA GC-3′ (forward) and 5′-TTC CAC AGA AAT ACA GTC CCT CTT G-3′ (Reverse). The PCR conditions were 98 °C for 30 s, followed by 35 cycles at 98 °C for 10 s, 64 °C for 30 s, and 72 °C for 47 s. The PCR products were then sequenced using a primer with the following sequence: 5′-GCC CCC TAC ATC GAC CAT TC-3′ (Macrogen). 

### 4.6. Transfection of the Expression Vectors

Cells were seeded into 6-well plates (4.5 × 10^5^ cells/well). The pcDNA3.0 empty vector, pcGNAQ, or pcGNAQ T96S were transfected into cells (2.5 μg/well) using Lipofectamine 2000 (Invitrogen) according to the manufacturer’s protocol. 

### 4.7. MTT Assay (Cell Viability Assay)

Cell proliferation was analyzed using a MTT assay (Amresco, Shanghai, China). The cells were then seeded into 96-well plates (4 × 10^3^ cells/well). At the appropriate time after transfection, 20 µL of MTT solution (5 mg/mL) was added to each well. After 3 h, media containing MTT solution were removed and 100 µL DMSO (Sigma-Aldrich, St. Louis, MO, USA) was added to each well. The absorbance at 540 nm was measured with a SoftMax apparatus (Molecular Devices, Sunnyvale, CA, USA). At least three independent experiments were performed to confirm the reproducibility of the results.

### 4.8. Soft Agar Colony Formation Assay

SK-Hep-1 cells were transfected with the control vector, pcGNAQ, or pcGNAQ T96S. After 24 h, the cells were suspended in the media containing 0.6% Bacto™ agar (214010, BD Difco, Franklin Lakes, NJ, USA) and overlaid on 1% agar in 6-well plates (2.5 × 10^3^ cells/well). After 3 weeks, colonies were photographed with a microscope and a camera. All visible colonies were counted. Four independent experiments were performed to confirm the reproducibility of the results.

### 4.9. Transwell Migration Assay

SK-Hep-1 cells were transfected with the control vector, pcGNAQ, or pcGNAQ T96S. After 24 h, the cells were suspended in a 100-μL serum-free medium and plated (1.3 × 10^4^ cells/well) into an 8.0-μm pore-size top chamber (Corning Life Sciences, Acton, MA, USA). The bottom chamber was filled with 700 μL of DMEM supplemented with 10% FBS as a chemo-attractant solution. The assembled chambers were placed in an incubator at 37 °C with 5% CO_2_ for 48 h. The chambers were then carefully washed with PBS, fixed with 100% methanol for 10 min, and stained with 0.1% crystal violet for 10 min. Cells remaining inside the top chamber were carefully removed with a cotton swab prior to microscopy. The numbers of migrated cells on the lower surface of the membranes were counted using a microscope (IX70, Olympus, Tokyo, Japan). Three independent experiments were performed to confirm the reproducibility of the results.

### 4.10. Western Blot 

Cells were lysed 24 h after transfection [42]. The cell lysate was subjected to 10% sodium dodecyl sulfate (SDS) polyacrylamide gel electrophoresis, and the separated proteins were transferred to a polyvinylidene fluoride membrane. Membranes were blocked and probed with the following antibodies: mouse anti-GNAQ, phospho-ERK (Santa Cruz, Dallas, TX, USA), rabbit anti-Flag, and ERK1/2 (Cell Signaling Technology, Beverly, MA, USA). The secondary antibodies were purchased from Santa Cruz. Protein bands were visualized using an enhanced chemiluminescence detection system (Amersham Bioscience, GE Healthcare, Piscataway, NJ, USA), and the membrane was exposed to X-ray film (Agfa, Mortsel, Belgium).

### 4.11. Genomic DNA Extraction and SNP Genotyping

Genomic DNA was extracted from Korean HCC tissue samples using the QIAmp DNA Blood Mini Kit (QIAGEN, Hilden, Germany) according to the manufacturer’s instructions; quality was checked using a spectrophotometer NanoDrop-2000 (Thermo Scientific, Wilmington, DE, USA). To test whether the GNAQ T96S mutation (rs753716491) found in TCGA-LIHC is also present in Korean patients, every DNA sample obtained from 456 HCC tissues was genotyped for the rs753716491 (https://www.ncbi.nlm.nih.gov/snp/, accessed date: 2 June 2020) using TaqMan^®^ analysis (Thermo Scientific). The real-time PCR reaction was conducted in a final volume of 8 µL, including 10 ng of genomic DNA and 3 µL of TaqMan^®^ Universal PCR Master Mix. Thermal cycling conditions were as follows: initial denaturing at 95 °C for 10 min, 50 cycles of 95 °C for 15 s, and 60 °C for 1 min. Genotyping was performed on a QuantStudio™ 6 Flex Real-Time PCR System (Thermo Scientific). Genotyping was repeated for all samples to confirm the results. We used the QuantStudio™ 6 Flex Real-Time PCR software version 1.2 (https://www.thermofisher.com/kr/ko/home/global/forms/life-science/quantstudio-6-7-flex-software.html, accessed date: 10 September 2020) for allelic discrimination. pcGNAQ and pcGNAQ T96S were used as controls.

### 4.12. In Silico Structure Analysis

The X-ray crystal structures of GNAQ complexes—including human RGS2 (PDB ID: 4EKD), human PLCbeta3 (phospholipase C-beta 3, 4GNK), GPCR kinase 2 (2BCJ), and p63RhoGEF (2RGN)—were obtained from the PDB databank (www.rcsb.org, accessed date: 20 December 2020). The structural visualization and inspection were performed using the software program Chimera [43]. Hydrogen atoms were added to entire models to identify hydrogen bonds. All of the residues near a specified single residue within 5 Å were selected and were analyzed for hydrogen bond formation.

## 5. Conclusions

Although HCC is caused by accumulated somatic mutations in various driver genes, the proposed molecular characterization of HCC cannot yet predict disease progression or recurrence [44,45]. In our study, GNAQ T96S expression increased HCC cell proliferation, anchorage-independent growth, and migration while also activating the MAPK signaling pathways. Previous reports show that an increase in the phosphorylated forms of ERK directly affects the phenotypes leading to metastasis or progression of HCC [46,47]. Thus, our data suggest that GNAQ T96S mutation may have enhanced oncogenic function of GNAQ in HCC via the MAPK signaling pathways.

## Figures and Tables

**Table 1:** Information on samples and patients having the guanine nucleotide-binding protein G(q) subunit alpha (GNAQ) T96S mutation in TCGA.

Tumor Sample	Matched Normal	Race	Age	Sex
CC-A8HS-01A-11D-A35Z-10	CC-A8HS-10A-01D-A35Z-10	Asian	18	M
DD-A4NF-01A-11D-A27I-10	DD-A4NF-10A-01D-A27I-10	White	72	M
DD-AACW-01A-11D-A40R-10	DD-AACW-10A-01D-A40U-10	Asian	43	M
DD-AADG-01A-11D-A40R-10	DD-AADG-10A-01D-A40U-10	Asian	70	M
DD-AADV-01A-11D-A38X-10	DD-AADV-10A-01D-A38X-10	Asian	50	M
DD-AADW-01A-11D-A38X-10	DD-AADW-10A-01D-A38X-10	Asian	48	M
DD-AAE8-01A-11D-A40R-10	DD-AAE8-10A-01D-A40U-10	Asian	45	M
DD-AAEK-01A-11D-A40R-10	DD-AAEK-10A-01D-A40U-10	Asian	51	M
ED-A97K-01A-21D-A382-10	ED-A97K-10A-01D-A385-10	Asian	54	M
G3-A3CG-01A-11D-A20W-10	G3-A3CG-10A-01D-A20W-10	White	80	M
K7-A5RF-01A-11D-A28X-10	K7-A5RF-10B-01D-A28X-10	White	64	M
MI-A75G-01A-11D-A32G-10	MI-A75G-10A-01D-A32G-10	White	63	M

## Data Availability

The data presented in this study are available upon request from the corresponding author.

## Supplementary Materials

The following are available online at https://www.mdpi.com/1422-0067/22/6/3284/s1, Figure S1: Supplementary data 1, Figure S2: Supplementary data 2.
